# Comparison study of bioelectrical impedance analyzers for measuring lower limb muscle mass in middle-aged and elderly adults

**DOI:** 10.3389/fnut.2025.1546499

**Published:** 2025-02-07

**Authors:** Ai-Chun Huang, Hsueh-Kuan Lu, Chien-Wei Liang, Kuen-Chang Hsieh, Yi-Sung Tsai, Chung-Liang Lai

**Affiliations:** ^1^Physical Education and Health Center, National Kaohsiung University of Hospitality and Tourism, Kaohsiung, Taiwan; ^2^General Education Center, National Taiwan University of Sport, Taichung, Taiwan; ^3^Office and Physical Education and Sport, National Chung Hsing University, Taichung, Taiwan; ^4^Department of Research and Development, Starbia Meditek Co., Ltd, Taichung, Taiwan; ^5^Big Data Center, National Chung-Hsing University, Taichung, Taiwan; ^6^Department of Physical Medicine and Rehabilitation, Taichung Hospital, Ministry of Health and Welfare, Taichung, Taiwan; ^7^Department of Physical Medicine and Rehabilitation, Puzi Hospital, Ministry of Health and Welfare, Chiayi, Taiwan; ^8^Department of Occupational Therapy, Asia University, Taichung, Taiwan

**Keywords:** sarcopenia, dual-energy X-ray absorptiometry, bioelectrical impedance, bioelectrical impedance analysis, skeletal muscle mass

## Abstract

**Objective:**

Lower limb muscle mass (LLMM) accounts for more than 50% of the total body skeletal muscle mass. Assessing leg muscle mass in middle-aged and elderly individuals is crucial for the prevention and diagnosis of sarcopenia. Current bioelectrical impedance analysis (BIA) devices are capable of measuring LLMM, but validation studies are limited. This study compares the accuracy of BIA devices with different frequencies for measuring LLMM in middle-aged and elderly populations.

**Methods:**

LLMM measurements were obtained using the following devices: foot-to-foot dual-frequency (StarBIA201, 5, 50 KHz), multi-segment single-frequency (Tanita BC418, 50 KHz), dual-frequency (InBody270, 20, 100 KHz), triple-frequency (Tanita MC780MA, 5, 50, 250 KHz), and six-frequency (InBody770, 1, 5, 50, 250, 500, 1,000 KHz). Dual-energy X-ray absorptiometry (DXA) served as the reference standard. Comparisons were conducted using the following metrics: (1) mean difference (bias), (2) limits of agreement (LOA), (3) Pearson correlation coefficients, and (4) ordinary least product (OLP) regression analysis.

**Results:**

A total of 153 community-dwelling individuals aged over 55 years (102 females, 51 males) were recruited. The average age of participants was 67.5 ± 8.9 years, with a BMI of 23.9 ± 3.9 kg/m^2^ and a body fat percentage of 35.8 ± 6.5%. The correlation coefficients of StarBIA201, BC418, InBody270, MC780, and InBody770 with DXA were 0.902, 0.903, 0.917, 0.925, and 0.928, respectively. Their mean differences were −0.141, −2.731, −0.587, −1.613, and −0.625 kg, with LOAs of 4.3, 5.7, 4.0, 5.1, and 3.8 kg, respectively. StarBIA201 and InBody270 showed no fixed or proportional biases.

**Conclusion:**

This study demonstrates that the four-electrode foot-to-foot BIA method shows significant practicality and potential in assessing LLMM. Compared to multi-frequency BIA and DXA, this method is simpler to operate and more convenient, making it particularly suitable for preliminary screening and assessment of sarcopenia in clinical and community settings.

## Introduction

Sarcopenia refers to age-related reductions in lean body mass, which affect the functional capacity of older adults (1). The prevalence of sarcopenia, adjusted for age and sex, ranges from 6 to 24%, depending on the definitions and measurement methods used for muscle mass assessment (2, 3).

Leg muscles are essential for supporting the body and performing daily activities. Insufficient low limb muscle mass (LLMM) in older adults can result in difficulties walking, unstable standing, and limitations in daily activities (4). This deficiency may also lead to accidents such as falls and fractures (5). The quantity and quality of LLMM influence basal metabolic rate and energy expenditure (6). Muscle loss may lower metabolic rates, increasing the risk of obesity and metabolic diseases (7). Insufficient LLMM may also contribute to cardiovascular health problems (7, 8). Adequate LLMM helps maintain the independence of older adults, enabling them to manage daily activities and sustain a good quality of life (9, 10).

Frailty is a syndrome commonly associated with reduced muscle mass and functional decline in older populations. It is closely related to sarcopenia and is a major factor contributing to mobility limitations, increased fall risk, and reduced quality of life (11). Research has highlighted the importance of early assessment and intervention for frailty in delaying the progression of sarcopenia (12). Existing studies suggest that the reduction in muscle mass, along with increased fat infiltration, significantly impacts lower limb functionality (13). However, most research focuses on total lean mass or appendicular skeletal muscle mass (ASMM), with limited studies specifically addressing LLMM (3). Dual-energy X-ray absorptiometry (DXA) is considered the gold standard for evaluating ASMM, but its high cost, operational complexity, and safety requirements make it more suitable for specific medical institutions (14). In contrast, bioelectrical impedance analysis (BIA), with its convenience, safety, and non-invasive nature, has gradually become a commonly used tool in clinical and community screening, particularly for the preliminary diagnosis of sarcopenia (15). However, the validity of BIA is often debated (16, 17). Numerous studies have evaluated the agreement between BIA and DXA, with some focusing on ASMM assessments (18, 19). However, these studies usually investigate lean mass of both upper and lower limbs combined, and there is limited research specifically exploring LLMM measurements using BIA compared to DXA in middle-aged and elderly populations.

Although numerous studies have explored the agreement between BIA and DXA in skeletal muscle mass measurements, these studies typically focus on total body or ASMM measurements (20). Furthermore, most studies have evaluated only single-frequency BIA devices, overlooking the potential of multi-frequency devices. This study not only compares various multi-frequency and design configurations of BIA devices but also systematically examines their performance in measuring LLMM, particularly under the influence of gender and BMI stratification. The objective of this study is to provide more targeted scientific evidence for selecting devices suitable for middle-aged and elderly people.

This study focuses on measuring LLMM in middle-aged and elderly individuals. It compares different types and frequencies of BIA devices and investigates their agreement with DXA measurements.

## Materials and methods

### Participants

The inclusion criteria for this study were community-dwelling older adults aged ≥55 years, with no history of nutritional disorders, endocrine disorders, or growth abnormalities, and body weight changes of less than 5 kg within 3 months prior to the study. Exclusion criteria included individuals using diuretics or medications that might affect fluid balance, those with recent surgeries or a history of major illnesses, and participants unable to stand for measurements.

All participants received detailed participant instructions 10 days before the experiment, requiring them to avoid diuretics for 7 days prior to testing and to abstain from alcohol 48 h before the test. Additionally, participants were instructed to urinate 20 min before the experiment to ensure stable body water levels. Fasting was not mandatory; however, participants were instructed to avoid drinking water or consuming foods with high water content at least 2 h before the measurement. Participants were also asked to refrain from having large meals, especially those high in salt or sugar, within 4 h prior to the measurement to avoid affecting body fluid distribution. All measurements were scheduled at 1:30 p.m. to minimize the influence of food intake on the results. Basic demographic information, including age, sex, and relevant health or medication history, was collected from all participants. After being briefed on the study procedures and their rights, participants signed informed consent forms and provided basic personal information. They wore cotton gowns (approximately 450 g). Measurements of physical parameters, BIA, and DXA were conducted sequentially within 90 min for each participant.

This study was conducted in compliance with the Declaration of Helsinki and approved by the Institutional Review Board (IRB) of the Ministry of Health and Welfare Nantou Hospital (IRB-112001, IRB-113002). The experiments were performed at Puzi Hospital, Ministry of Health and Welfare, Chiayi County, Taiwan, between June 2023 and December 2024.

### Anthropometric measurements

Height was measured using a stadiometer (Holtain, Crosswell, Wales, United Kingdom) to the nearest 0.5 cm with participants standing barefoot. Body weight was measured using calibrated weight scales integrated into the BIA devices. Body mass index (BMI) was calculated as weight (kg) divided by height (m^2^), with units expressed as kg/m^2^.

### Bioelectrical impedance analysis (BIA)

Participants wore the provided cotton clothing for the measurements. Five types of BIA devices were used to measure LLMM, and the detailed device information is summarized in Table 1. The input parameters for each device included the participant’s gender, age, height, and weight. All measurements were conducted with participants standing barefoot, and the corresponding LLMM was calculated based on the characteristics of each device.

**Table 1:** Summary of BIA devices used in the study.

Device name	Model	Measurement frequency (KHz)	Inputs required	Measured parameters*
StarBIA201	Foot-to-Foot	5, 50	Age, Sex, Height	a, b, c, d, e, f, g, h, i, m
Tanita BC418	Multi-Segment	50	Age, Sex, Height	a, b, i, j, k, l, m, u, v
InBody270	Multi-Segment	20, 100	Age, Sex, Height	a, b, c, d, i, j, k, l, m, n, o, p, q, r, s, t, u, v
Tanita MC780	Multi-Segment	5, 50, 250	Age, Sex, Height	a, b, c, d, h, i, j, k, l, m, h, u, v, w, x
InBody770	Multi-Segment	1, 5, 50, 250, 500, 1,000	Age, Sex, Height	a, b, c, d, h, i, j, k, l, m, n, o, p, q, r, s, t, u, v, w, x, y, z

*a: weight, b: body fat percentage, c: bone mineral content, d: visceral fat area, e: appendicular lean mass index, f: lower limbs muscle mass, g: bone mineral density, h: ECW/TBW ration, i: basal metabolic rate, j: Total body water, k: segmental fat (right arm, left arm, trunk, right leg, left leg), l: segmental lean (right arm, left arm, trunk, right leg, left leg), m: BMI, n: protein, o: muscle mass, p: waist-hip ration, q: waist circumference, r: Inbody score, s: fat control, t: muscle control, u: fat mass, v: free fat mass, w: ECW, x: ICW, y: segmental body water (right arm, left arm, trunk, right leg, left leg), z: Segmental ECW Ration (right arm, left arm, trunk, right leg, left leg).

### Dual-energy X-ray absorptiometry (DXA)

Participants first underwent BIA, with measurements scheduled between 2:00 PM and 4:00 PM. To ensure consistency in the measurement results, all participants completed both BIA and DXA measurements on the same day. Immediately after finishing the BIA measurement, participants proceeded to the DXA measurement room for DXA. All DXA measurements were completed by 5:30 PM.

The Lunar Prodigy DXA system (GE Healthcare, United States) was used to measure total body fat mass, fat percentage, and lean soft tissue mass. Participants wore lightweight cotton gowns and lay relaxed in a supine position on the DXA scanner bed, with arms extended alongside their body and feet together with toes pointing upward. The whole-body scan mode was employed, taking approximately 10 min per participant.

The enCORE 2003 Version 7.0 software automatically calculated body composition for total body, trunk, android, gynoid, upper limbs, and lower limbs.

## Statistical analysis

All data were expressed as means ± standard deviations (SDs) and ranges. Normal distribution was assessed using the Kolmogorov–Smirnov test. Statistical analyses were conducted using SPSS Version 20 (IBM SPSS Statistics for Windows, Armonk, NY), with significance set at *p* < 0.05 (two-tailed).

Paired t-tests were used to compare LLMM measurements obtained from DXA and BIA devices. Correlations between BIA estimates and DXA measurements were assessed using Pearson’s correlation coefficients and ordinary least products (OLP) regression analysis to determine proportional bias and fixed error (21). Linear regression analysis provided correlation coefficients (r), determination coefficients (r^2^), and the standard error of the estimate (SEE) to evaluate prediction accuracy and consistency between devices.

The degree of agreement between BIA and DXA was evaluated using Bland–Altman plots, intraclass correlation coefficients (ICC), and Lin’s concordance correlation coefficients (CCC). ICC (r1) with two-way random and single-measure models assessed consistency, with r1 ≥ 0.8 indicating high consistency (22). CCC ($\rho_{C}$) evaluated the closeness of fit between measurements and a 45-degree reference line, with $\rho_{C}$ values classified as follows: almost perfect ($\rho_{C}$ > 0.99), substantial (0.99 ≥ $\rho_{C}$ > 0.95), fair (0.95 ≥ $\rho_{C}$ ≥ 0.9), and poor ($\rho_{C}$ < 0.9) (23).

Bland–Altman plots displayed differences between measurements and averages, with limits of agreement (LOA) used to assess consistency for identical variables (24). Regression lines illustrated the trends of differences between devices.

Consider conducting a correlation analysis using the software G*Power Ver 3.1.94. Under the conditions where the *α* err prob. is set at 0.05 and the power (1−*β* err prob) is set at 0.95, the minimum required sample size is 138.

Participants were classified into two groups based on the Asian Working Group for Sarcopenia (AWGS) diagnostic criteria for sarcopenia: the sarcopenia group (handgrip strength <28 kg for men and < 18 kg for women; appendicular skeletal muscle mass index <7.0 kg/m^2^ for men and < 5.7 kg/m^2^ for women) and the non-sarcopenia group. The consistency between BIA and DXA measurements was analyzed in each group.

## Results

In this study, a total of 153 eligible participants completed the measurements, including 102 females (66.7%) and 51 males (33.3%). The average age of participants was 67.5 ± 8.9 years, and the average BMI was 23.9 ± 3.9 kg/m^2^. The LLMM of males (14.6 ± 2.9 kg) was significantly higher than that of females (10.6 ± 1.5 kg) (*p* < 0.001). Participant demographics and body composition data are presented in Table 2. Male participants had a slightly higher BMI (24.7 ± 2.9 kg/m^2^) compared to females (23.7 ± 4.0 kg/m^2^). According to DXA measurements, males had a lower body fat percentage (28.9 ± 4.0%) than females (37.5 ± 5.8%). Male LLMM was 14.6 ± 2.9 kg, significantly higher than that of females (10.6 ± 1.5 kg). Male LLMM, tissue mass, bone mineral content, and bone mineral density (BMD) were significantly higher than females (*p* < 0.001).

**Table 2:** Anthropometric characteristics and body composition measurements of older determined by DXA (reference method).

	All (*n* = 153)			Female (*n* = 102)		Male (*n* = 51)		
	Mean ± SD		Min-max	Mean ± SD	Min-max	Mean ± SD	Min-max	*p*
Age (yrs)	67.5 ± 8.9		55, 91	66.7 ± 8.7	55, 91	72.4 ± 9.8**	55, 90	0.0010
Height (cm)	157.1 ± 6.3		143, 178	155.7 ± 4.6	143, 168	165.5 ± 6.5**	152, 178	9.5E-17
Weight (kg)	59.7 ± 11.2		35.0, 94.9	57.5 ± 9.9	35.0, 94.9	68.2 ± 11.4**	47.6, 87.0	3.9E-7
BMI (kg/m^2^)	23.9 ± 3.9		13.3, 39.5	23.7 ± 4.0	13.3, 39.5	24.7 ± 2.9	19.0, 30.3	0.1072
PBF_DXA_ (%)	35.8 ± 6.5		19.3, 48.7	37.5 ± 5.8	19.3, 48.7	28.9 ± 4.0**	23.1, 40.6	1.3E-12
LLLM_DXA_ (kg)	11.4 ± 2.5		5.5, 18.9	10.6 ± 1.5	5.49, 15.1	14.6 ± 2.9**	8.8, 18.8	9.3E-10
LBMC_DXA_ (kg)	0.64 ± 0.15		0.39, 1.11	0.59 ± 0.09	0.39, 0.87	0.83 ± 0.15**	0.55, 1.11	2.7E-21
LFM_DXA_ (kg)	6.7 ± 2.8		3.0, 18.9	6.9 ± 2.3	3.0, 18.9	6.10 ± 1.38*	3.24, 7.85	0.073
LTissue_DXA_ (kg)	18.3 ± 3.9		12.3, 29.7	17.7 ± 3.6	12.3, 29.7	28.9 ± 4.0**	23.1, 40.6	0.0001
LBMD_DXA_ (g/cm^2^)	1.03 ± 0.15		0.74, 1.68	0.99 ± 0.14	0.74, 1.67	1.18 ± 0.12**	0.95, 1.43	2.1E-10

BMI, Body Mass Index; DXA, dual-energy X-ray absorptiometry; PBF, percent body fat; LBMC, Bone Mineral Content of Legs; LLLM, Lower Limbs Muscle Mass; LFM, Fat mass of Legs; LTissue, Tissue of Legs; LBMD, Bone Mineral Density of Legs.

^*^p < 0.05, by repeated-measures ANOVA with Student’s Independent *t*-test.

^**^p < 0.01, by repeated-measures ANOVA with Student’s Independent *t*-test.

Table 3 summarizes the results of LLMM measurements using different BIA devices compared to DXA. The mean measurement of StarBIA201 (10.3 ± 2.4 kg) was significantly lower than that of DXA (11.4 ± 2.5 kg). The BC418 device reported higher mean values (14.1 ± 3.2 kg) with greater bias compared to DXA. Measurements from InBody270 and InBody770 (both approximately 12.1 kg) were closer to DXA, while MC780 reported higher mean values (13.0 ± 3.1 kg) than DXA but lower than BC418.

**Table 3:** Results of lower limb muscle mass measured by different bioelectrical impedance body composition devices and DXA.

Device	All (*n* = 153)		Female (*n* = 102)		Male (*n* = 51)		
	Mean ± SD	Min–max	Mean ± SD	Min–max	Mean ± SD	Min-max	*p*
StarBIA210 (kg)	10.3 ± 2.4	6.8, 17.8	9.5 ± 1.3	6.8, 13.8	12.1 ± 2.9^**^	8.4, 18.9	2.4E-11
BC418 (kg)	14.1 ± 3.2	9.8, 25.1	12.8 ± 1.5	9.8, 18.1	16.9 ± 3.9^**^	11.5, 25.1	3.6E-15
Inbody270 (kg)	12.1 ± 2.4	7.7, 19.6	11.3 ± 1.5	7.7, 15.5	13.8 ± 3.0^**^	9.8, 19.6	3.1E-10
MC780 (kg)	13.0 ± 3.1	8.0, 23,4	12.0 ± 1.6	8.0, 18.2	15.5 ± 3.8^**^	10.3, 23.4	2.1E-11
Inbody770 (kg)	12.1 ± 2.5	8.1, 19.8	11.2 ± 1.5	8.1, 16.2	13.9 ± 3.2^**^	8.4, 19.8	1.7E-10
DXA (kg)	11.4 ± 2.5	5.5, 18.9	10.6 ± 1.5	5.49, 15.1	14.6 ± 2.9^**^	8.8, 18.8	9.3E-10

^**^p < 0.01, by repeated-measures ANOVA with Student’s Independent *t*-test.

Table 4 presents the correlation and agreement between different BIA devices and DXA measurements of LLMM across gender and BMI categories. Results showed that correlation coefficients for all devices ranged from 0.8 to 0.96, indicating moderate to high linear correlations. InBody770 and InBody270 demonstrated the highest correlations (r ≥ 0.950), particularly in males and the high BMI groups (overweight and obese). StarBIA201 and InBody770 exhibited the narrowest LOA (approximately −0.97 to 2.52 kg and −1.35 to 2.56 kg, respectively), indicating better agreement with DXA. In contrast, BC418 and MC780 showed the widest LOA, particularly in the high BMI group, suggesting greater variability in measurements. Male participants generally exhibited higher correlation and agreement than females, especially with InBody270 and InBody770, where male measurement bias was generally lower. The normal BMI group demonstrated the smallest bias and LOA, indicating more stable measurement results.

**Table 4:** Agreement between BIA devices and DXA by sex and BMI categories.

Device	Group*	r	Mean difference (kg)	LOA (kg)
StarBIA201	Male (*n* = 51)	0.94	−1.03 ± 1.02	−3.08 to 1.02
	Female (*n* = 102)	0.83	−1.16 ± 1.10	−3.37 to 1.05
	Normal BMI (*n* = 96)	0.88	−0.75 ± 0.93	−2.61 to 1.11
	Overweight BMI(*n* = 44)	0.94	−1.71 ± 0.90	−3.50 to 0.09
	Obesity BMI (*n* = 13)	0.94	−2.16 ± 1.09	−4.34 to 0.03
BC418	Male (*n* = 51)	0.92	3.82 ± 1.60	0.62 to 7.03
	Female (*n* = 102)	0.80	2.24 ± 0.95	0.35 to 4.14
	Normal BMI (*n* = 96)	0.84	2.65 ± 1.36	−0.06 to 5.37
	Overweight BMI(*n* = 44)	0.93	2.87 ± 1.43	0.0 to 5.74
	Obesity BMI (*n* = 13)	0.92	2.81 ± 1.80	−0.78 to 6.41
Inbody270	Male (*n* = 51)	0.95	0.71 ± 1.00	−1.30 to 2.73
	Female (*n* = 102)	0.82	0.71 ± 0.98	−1.25 to 2.67
	Normal BMI (*n* = 96)	0.88	0.95 ± 0.93	−0.91 to 2.81
	Overweight BMI (*n* = 44)	0.94	0.32 ± 0.90	−1.48 to 2.12
	Obesity BMI (*n* = 13)	0.95	0.0 ± 0.99	−1.99 to 1.97
MC780	Male (*n* = 51)	0.95	2.40 ± 1.23	−0.09 to 4.85
	Female (*n* = 102)	0.81	1.29 ± 1.14	−0.99 to 3.57
	Normal BMI (*n* = 96)	0.85	1.63 ± 1.16	−0.69 to 3.97
	Overweight BMI (*n* = 44)	0.94	1.44 ± 1.33	−1.22 to 4.11
	Obesity BMI (*n* = 13)	0.96	2.02 ± 1.85	−1.54 to 5.86
Inbody770	Male (*n* = 51)	0.96	0.77 ± 0.87	−0.97 to 2.52
	Female (*n* = 102)	0.87	0.61 ± 0.97	−1.35 to 2.56
	Normal BMI (*n* = 96)	0.90	0.87 ± 0.89	−0.93 to 2.66
	Overweight BMI (*n* = 44)	0.95	0.23 ± 0.84	−1.45 to 1.91
	Obesity BMI (*n* = 13)	0.96	0.15 ± 0.89	−1.63 to 1.93

^*^Normal BMI: BMI ≤ 25 kg/m^2^, Overweight BMI: 25 kg/m^2^ < BMI ≤ 30 kg/m^2^; Obesity BMI: 30 kg/m^2^ < BMI.

Table 5 shows the Pearson product–moment correlation coefficients (r) and linear regression equations between LLMM estimates from BIA devices and DXA. All five BIA devices examined in this study showed a strong linear correlation with DXA measurements (r ≥ 0.9 for all BIA devices). However, except for the StarBIA201, other BIA devices exhibited proportional bias and/or fixed bias.

**Table 5:** Correlation of lean mass estimates using Pearson product moment correlation and ordinary least product regression.

Device	r	a	95%CI	b	95%CI	Fixed bias	Proportional bias	RSD
StarBIA201	0.902	−0.800	−1.953, 0.149	1.079	0.991, 1.181	No	No	1.082
BC418MA	0.903	1.527^**^	0.752, 2.303	0.699	0.646, 0.752	Yes	Yes	1.074
Inbody270	0.917	−0.033	−0.849, 0.780	0.946	0.880, 1.021	No	No	0.998
MC780	0.925	1.730^**^	0.995, 2.465	0.734	0.689, 0.798	Yes	Yes	1.044
InBody770	0.928	0.262	−0.475, 1.001	0.926^**^	0.866, 0.986	No	Yes	0.935

r: Pearson product moment correlation coefficient; a, b coefficients in ordinary least products regression model: $E\left(A\right)=a+b\left(B\right)$; a (y axis) intercept; b, slope; fixed bias, if 95% confidence interval (CI) for a does not include 0; proportional bias, if 95% confidence interval (CI) for b does not include 1; RSD, Residual Standard Deviation. **p< 0.01.

While Pearson correlation quantifies the strength of the linear relationship between two methods for the same variable, it is not ideal for evaluating agreement between methods. Therefore, three additional statistical techniques were employed to assess agreement between BIA devices and DXA for estimating LLMM: Bland–Altman plots, CCC, and ICC, as shown in Table 6. ICC values (r1 ≥ 0.8) indicated high consistency between BIA devices and DXA. CCC values for the BIA devices varied, ranging from 0.603 to 0.899 in this study.

**Table 6:** Agreement between bioelectrical impedance analysis and dual-energy X-ray absorptiometry.

Device	Bland–Altman plot						
	Bias	LOA	Trend line	CCC($\rho_{C})$	Cb	Cu	ICC(r_1_)
StarBIA201	−0.141	−2.271, 1.982	y = 0.480−0.054 x	0.811	0.898	*p* > 0.05	0.908
BC418MA	−2.731	−5.654, 0.101	y = 0.690−0.267^*^x	0.603	0.667	*p* < 0.05	0.874
Inbody270	−0.687	−2.653, 1.279	y = −1.066^*^ + 0.032 x	0.882	0.962	*p* > 0.10	0.917
MC780	−1.613	−4.166, 0.938	y = 0.951−0.209^**^x	0.762	0.839	*p* > 0.05	0.891
InBody770	−0.625	−2.487, 1.237	y = −0.607−0.001 x	0.899	0.969	*p* > 0.10	0.927

LOA, limits of agreement; CCC, Lins concordance correlation coefficient; Cb, Bias correction factor (accuracy); Cu, Cusum test for linearity; ICC, Interclass correlation. *p< 0.05; **p<0.01.

**Figure 1:**
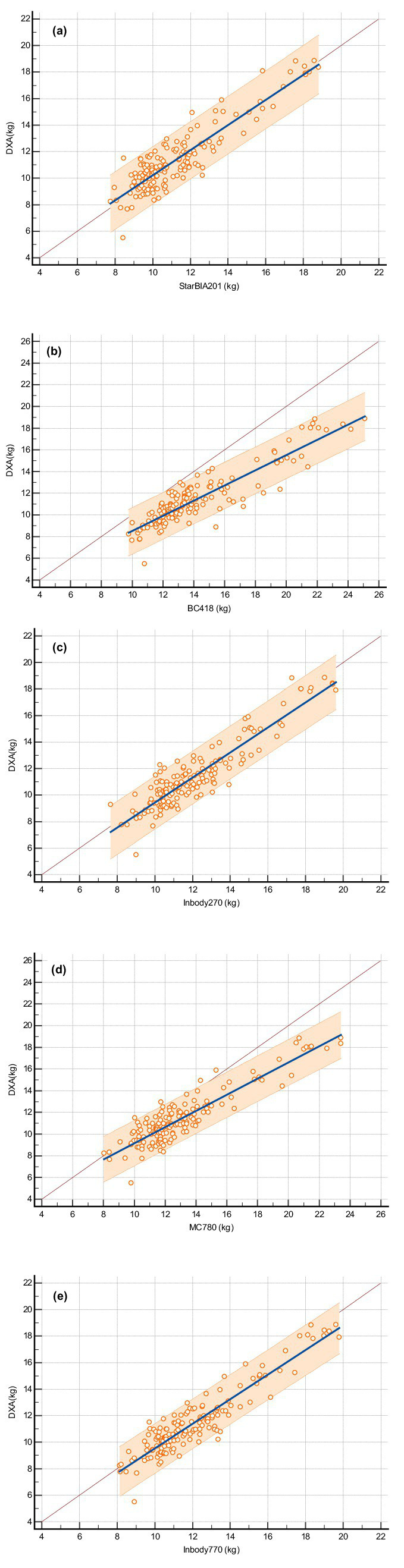
Scatter diagrams and regression lines for lower limb muscle mass measurements using bioelectrical impedance analysis (BIA) devices and DXA. **(A)** StarBIA201, **(B)** BC418, **(C)** InBody270, **(D)** MC780, **(E)** InBody770. The figure displays the regression line (solid line), the confidence interval for the regression line (dashed lines), and the identity line (x = y, dotted line).

Figure 1 shows scatter plots and regression lines for LLMM measurements obtained from BIA devices and DXA. The regression lines for some BIA devices deviated significantly from the equivalence line, particularly in Figures 1B,D, indicating that BIA devices overestimated LLMM compared to DXA.

**Figure 2:**
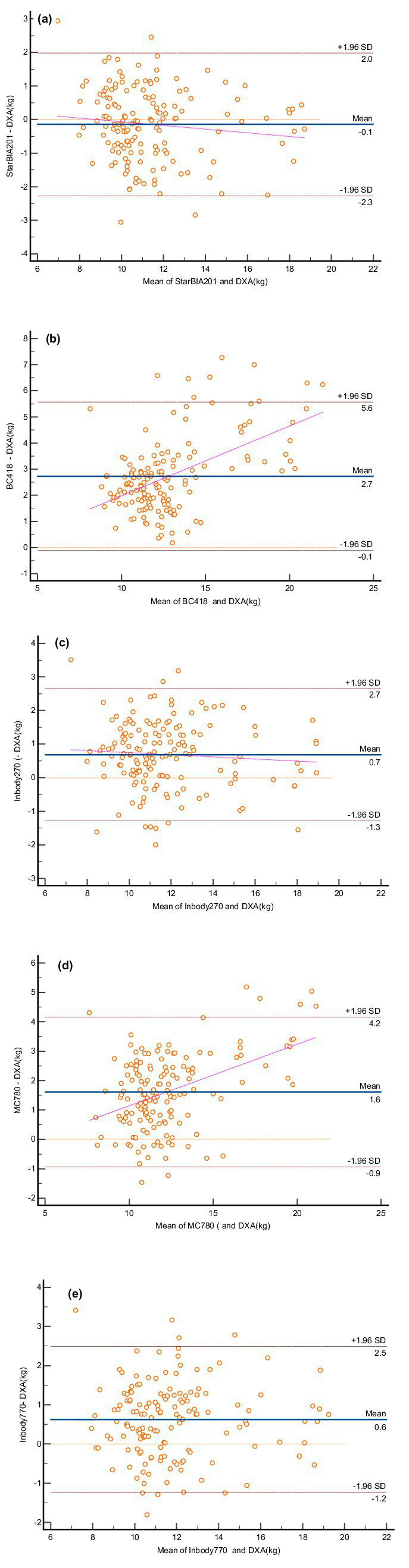
Bland–Altman plots for lower limb muscle mass measurements using bioelectrical impedance analysis (BIA) devices and DXA. **(A)** StarBIA201, **(B)** BC418, **(C)** InBody270, **(D)** MC780, **(E)** InBody770. The blue solid line represents the mean difference between the two methods, and the dotted lines indicate the 95% confidence intervals for the upper and lower limits.

Figure 2 presents Bland–Altman plots illustrating bias and limits of agreement (LOA) between BIA and DXA measurements. Figures 2B,D show that the BC418 and MC780 devices overestimated LLMM by 2.7 kg and 1.6 kg, respectively, compared to DXA. The StarBIA201 underestimated LLMM by 0.1 kg, while the InBody270 and InBody770 overestimated it by 0.7 kg and 0.6 kg, respectively. Notably, Figures 2B,D also revealed evident proportional bias in the measurement differences for the BC418 and MC780 devices.

In the sarcopenia group (*n* = 40), StarBIA201 showed the highest correlation with DXA (r = 0.91), with a bias of −0.15 ± 1.2 kg and LOA of −2.3 to 2.0 kg. In the non-sarcopenia group (*n* = 113), six-frequency multi-segmental BIA devices (e.g., InBody770) exhibited higher agreement (r = 0.94, LOA = −1.8 to 1.2 kg).

## Discussion

The primary objective of this study was to evaluate the consistency and accuracy of various BIA devices compared with DXA in measuring LLMM among older adults. Since LLMM plays a crucial role in the early diagnosis of sarcopenia, the findings of this study provide valuable references for clinical and community-based screening. Reduction in LLMM is a core characteristic of sarcopenia, directly impacting walking speed, balance, and mobility, increasing the risk of falls, and limiting activities of daily living (ADL). This study demonstrates that all tested BIA devices were capable of detecting changes in LLMM, particularly StarBIA201 and InBody270, which excelled in terms of low cost and high convenience. These findings offer clinicians a practical option for the early screening and diagnosis of sarcopenia. Using Pearson correlation is useful for testing linear relationships, Bland–Altman plots are better suited for evaluating agreement between BIA devices and DXA. However, the best method for assessing agreement between two tools remains unclear. CCC and ICC provide scaled indices of agreement based on measurement ranges, making them easy to summarize but harder to interpret (25). In contrast, the bias and LOA from Bland–Altman plots are unscaled indices based on raw measurement ranges and require understanding of the measured variable for interpretation (26). Given the limitations of each method, multiple statistical approaches were used in this study to evaluate agreement between the devices.

Reduction in LLMM is a key feature of sarcopenia, directly impacting walking speed, balance, and mobility (27). Decreased LLMM increases the risk of falls and mobility impairments, which can affect daily living activities (ADL) (28). Although most studies refer to ASMM as a comprehensive indicator, specific methods for measuring LLMM, such as magnetic resonance imaging (MRI) or computed tomography (CT) for assessing quadriceps cross-sectional area, are recommended for early sarcopenia diagnosis. DXA and BIA provide practical and convenient methods for estimating LLMM in clinical and research settings (29).

Research on the use of BIA to measure total lean mass or ASMM has been extensive. Studies have consistently shown a strong correlation (r > 0.9) (30) between ASMM measurements obtained using multi-segmental BIA devices, such as the InBody770, and DXA. In this study, all five BIA devices evaluated (StarBIA201, BC418, MC780, InBody270, and InBody770) also demonstrated high correlations (r > 0.9) with DXA in assessing LLMM. Lee et al. (30) reported that multi-segmental BIA devices overestimated ASMM by 2.3 kg in males and 1.8 kg in females. Additionally, their study found that the discrepancy between BIA and DXA measurements was larger in low BMI groups, with age showing no significant effect on ASMM differences. Similarly, Montgomery et al. (31) observed that the InBody770 underestimated LLMM by 1.5 kg in females and 3.0 kg in males, with consistency ranges (LOA) of 4.9 kg and 4.2 kg, respectively. In our study, the LOA range for the InBody770 was 3.7 kg, which aligns closely with Montgomery et al.’s findings when considering the average weight of our participants. Furthermore, NcLester et al. (32) compared the accuracy of the InBody230, InBody720, and InBody770 for estimating total fat-free mass (FFM) and found all three models to overestimate FFM, with minimal differences between them. The precision of the InBody270 and InBody770 for LLMM observed in our study aligns with the trends reported by NcLester et al. (32), who observed overestimation of FFM across InBody230, InBody720, and InBody770, with minimal differences among models. The InBody270 and InBody770 also showed minimal differences in LLMM estimation in this study.

To more accurately evaluate the agreement between BIA and DXA, this study utilized Bland–Altman analysis, correlation coefficients (r), and concordance correlation coefficients (CCC). Results showed that StarBIA201 and InBody270 exhibited high consistency with DXA measurements (r > 0.9, LOA: −2.3 to 1.9 kg), with no significant fixed or proportional bias. This indicates their potential for clinical application, especially in resource-limited settings. The results of this study reveal differences in the consistency between BIA and DXA measurements in the sarcopenia and non-sarcopenia groups. Multi-segmental BIA devices performed better in the non-sarcopenia group compared to the sarcopenia group. Additionally, the findings for StarBIA201 in the sarcopenia group suggest its suitability as a preliminary screening tool for sarcopenia. Future research should explore device calibration methods tailored for specific populations, such as individuals with severe sarcopenia.

The algorithms used by different BIA devices may be developed based on varying datasets. For instance, some devices (such as the InBody series) utilize DXA measurement data to construct their models, enhancing the accuracy of lean mass estimation. In contrast, other devices (such as StarBIA201) may rely on internal calibration datasets or alternative methodologies. Due to commercial reasons, the detailed algorithms of certain devices remain undisclosed, potentially affecting the consistency of result comparisons. In this study, all BIA devices demonstrated high correlations with DXA measurements of LLMM (r ≥ 0.9). This consistency can be partially attributed to the fact that some device algorithms were developed using DXA data, ensuring alignment between BIA and DXA results in lean mass measurements. However, our study further revealed biases in different devices across gender and BMI subgroups, suggesting that even with algorithms based on the same DXA data, device design and calibration methods can influence performance. This study highlights that the agreement between BIA and DXA results may partially stem from the foundational data of the algorithms. However, such agreements do not guarantee the applicability of all devices across all populations. Future studies should further validate device performance under specific conditions, such as obesity, older populations, or unique pathological conditions.

All BIA measurements in this study were conducted with participants in a standing position. Participants stood barefoot on the foot electrodes of the measurement device and, where applicable, held the hand electrodes. This posture aligns with the operational standards of most BIA devices and is more representative of practical applications in clinical and community screening settings. In this study, BIA measurements were performed in the standing position, while DXA measurements were conducted in the supine position. Postural differences may influence body fluid distribution, thereby affecting impedance measurements. Specifically, in the supine position, body fluids may redistribute towards the upper body, potentially introducing bias into the results (33).

One significant advantage of multi-frequency BIA is its ability to simultaneously measure intracellular water (ICW) and extracellular water (ECW), offering the potential for a more comprehensive evaluation of fluid distribution (34). This study primarily focused on the measurement of LLMM and therefore did not further analyze the correlation between the ECW/ICW ratio and DXA. However, existing literature indicates that abnormalities in fluid distribution may be associated with sarcopenia and other metabolic diseases (35). Future studies should consider incorporating the ECW/ICW ratio into the analysis to explore its clinical relevance.

Multi-segmental BIA is a convenient method for estimating segmental muscle mass compared to DXA. Foot-to-foot BIA analyzers offer even greater convenience for measuring LLMM. However, future work should focus on establishing healthy ranges for LLMM based on population demographics. Standardized methods, such as using mean ± 2 standard deviations or setting a T-score ≤ −2.5 as a threshold for low muscle mass, should be adopted. Localized or ethnicity-specific standards should also account for differences in body composition, such as the generally lower muscle mass in Asian populations compared to Europeans (36). Establishing standards for LLMM using DXA-calibrated equations, such as the proportion of LLMM to total body muscle mass, may provide a more practical approach for sarcopenia assessment and prevention.

Existing methods for measuring muscle mass include central or peripheral quantitative computed tomography (QCT), which measures muscle cross-sectional area and muscle density as an indicator of fat infiltration; MRI, which measures muscle cross-sectional area and volume; and BIA, which estimates fat-free mass, total body, and segmental muscle mass. Objective measures of physical function are widely applied in sarcopenia research, including grip strength, lower extremity strength, and gait speed tests. Among these, short-distance walking speed (3–6 m) is the most commonly used objective physical function assessment method in research and is gradually becoming a standard approach in clinical evaluation (37).

In summary, various methods are available for assessing muscle mass and physical function, each with its advantages and limitations. The choice of method depends on the nature of the research question, the clinical setting, and the resources available. Grip strength testing, as an indicator of upper extremity strength, is widely used in both research and clinical contexts. However, lower extremity strength is likely more relevant in studies focusing on mobility. Due to the complexity and cost of measuring lower extremity strength, it is less frequently incorporated into integrated sarcopenia definitions. Research indicates that lower extremity strength measurements, such as gait speed and lower extremity strength tests, are better reflections of mobility status and are significantly associated with lower extremity muscle mass. Therefore, the assessment of lower extremity muscle mass is particularly necessary (29, 38).

The results of this study demonstrate that multi-frequency BIA devices (such as StarBIA201 and InBody270) exhibit higher consistency in measuring LLMM (r ≥ 0.9) compared to single-frequency devices, with no fixed or proportional bias. This is consistent with previous reports on ASMM measurements (39). However, our study is the first to highlight that gender and BMI significantly influence the measurement accuracy of certain devices for LLMM, particularly showing increased bias in the obese group. These findings extend the applicability of previous research and provide new perspectives for personalized assessment.

This study highlights that the agreement between BIA and DXA results may partially stem from the foundational data of the algorithms. However, such consistency does not guarantee the applicability of all devices across all populations. Future studies should validate device performance under specific conditions, such as obesity, older populations, or unique pathological conditions.

## Limitations

This study has several limitations. First, it is not community-based and used non-probabilistic convenience sampling. Second, all participants were middle-aged and elderly individuals from Taiwan, limiting the generalizability of the findings to other populations or the general public, and requiring further cross-validation. Third, most companies selling BIA devices do not disclose the formulas used for muscle mass estimation, restricting their use for research purposes. Fourth, All measurements in this study were conducted in a standing position, making them unsuitable for participants unable to stand, such as amputees or bedridden individuals. Furthermore, the impedance measurement method used in BIA relies on uniform body water distribution, which may be affected in amputees. Future research should explore improved methods or alternative technologies tailored to these populations.

## Conclusion

This study found slight differences in the performance of multi-segmental BIA devices when measuring data from males and females, particularly in populations with obesity, where some devices exhibited notable biases. This suggests that differences in gender and body composition characteristics may influence the measurement accuracy of the devices. Future research should further explore optimized measurement algorithms tailored to specific populations.

The findings of this study not only provide scientific evidence for selecting appropriate BIA devices for different settings but also offer specific guidance for the early diagnosis of sarcopenia. Future studies should include cross-cultural validation across diverse populations to further extend the applicability and generalizability of the research.

## Data Availability

The raw data supporting the conclusions of this article will be made available by the authors, without undue reservation.
